# Exosome-mediated miRNA delivery: a molecular switch for reshaping neuropathic pain therapy

**DOI:** 10.3389/fnmol.2025.1625943

**Published:** 2025-07-04

**Authors:** Ziqing Wei, Chunhui Guo, Hang Zhou, Yanling Wu, Xudong Zhou, Jibing Chen, Fujun Li

**Affiliations:** ^1^Graduate School, Guangxi University of Chinese Medicine, Nanning, China; ^2^Ruikang Hospital Affiliated to Guangxi University of Chinese Medicine, Nanning, China; ^3^Graduate School, Guangxi University, Nanning, China; ^4^Zhuhai Maternal and Child Health Care Hospital, (Guangxi University of Chinese Medicine), Zhuhai, China

**Keywords:** neuropathic pain, targeted delivery, miRNA therapeutics, exosomes, pain management, clinical translation

## Abstract

Neuropathic pain (NP) is a chronic condition caused by nerve injury or disease. It remains a therapeutic challenge because conventional drugs have limited efficacy and cause adverse effects. Exosomes, with the ability to cross the blood-brain barrier, low immunogenicity, and tissue-homing capacity, have emerged as promising nanovehicles for precise microRNA (miRNA) delivery to modulate key NP pathologies such as neuroinflammation, neuronal hyperexcitability, mechanical allodynia, and thermal hyperalgesia. In this review, we highlight recent advances in exosome-mediated miRNA therapy for NP. We also elucidate the molecular mechanisms and unique advantages of exosomes as both delivery platforms and intrinsic therapeutic agents. We synthesize evidence from preclinical models and initial clinical-stage studies, addressing translational challenges in scalable production and targeted delivery. Through sustained innovation and multidisciplinary collaboration, exosome-based miRNA delivery systems demonstrate transformative potential to overcome current therapeutic limitations, enabling novel NP management strategies.

## 1 Introduction

Neuropathic pain (NP) constitutes a debilitating chronic condition originating from lesions or diseases affecting the somatosensory nervous system (Finnerup et al., 2021). Afflicting approximately 7% of the global population, it imposes substantial clinical, economic, and societal burdens (Savelieff et al., 2025). Patients typically experience characteristic manifestations including spontaneous burning sensations, electric shock-like pain, and mechanically evoked allodynia—paradoxical pain perception in response to non-noxious stimuli such as light touch. The pathophysiology involves multifaceted mechanisms encompassing peripheral and central sensitization (e.g., dysregulated ion channels, NMDA receptor activation), neuroinflammation (e.g., TLR4/NF-κB signaling in glial cells), and impaired neural repair (Vranken, 2012; Zhang et al., 2023; Kaye et al., 2024).

Current NP management primarily relies on pharmacological interventions (e.g., antidepressants, calcium channel modulators, topical anesthetics) (Alcántara-Montero and Pacheco-de Vasconcelos, 2022; Bayer et al., 2004; Moisset et al., 2022) and neuromodulation techniques [e.g., transcutaneous electrical nerve stimulation (TENS), spinal cord stimulation (SCS)] (da Silva et al., 2024; Leung et al., 2020). However, these approaches face critical limitations: systemic adverse effects (sedation, anticholinergic effects), insufficient efficacy in subsets of patients, and inability to concurrently target multiple NP mechanisms. Compromised central nervous system bioavailability due to the blood-brain barrier (BBB) further restricts therapeutic effectiveness (Amescua-Garcia et al., 2018; Ardeleanu et al., 2020). Consequently, NP remains inadequately managed in many patients, underscoring the urgent need for novel multitargeted therapeutic strategies with enhanced central nervous system (CNS) delivery capabilities.

MicroRNAs (miRNAs), serving as pivotal post-transcriptional regulators in NP pathogenesis, modulate neuroinflammation, neuronal hyperexcitability, and nerve repair (Wang et al., 2024). Their capacity to concurrently fine-tune multiple target gene networks offers significant therapeutic advantages over single-target drugs (Chang et al., 2017; Peng et al., 2017). Nevertheless, clinical translation of miRNA therapies is hindered by rapid degradation, poor cellular uptake, and inefficient BBB penetration (Shityakov et al., 2022). Exosomes—natural nanoscale extracellular vesicles—provide a promising solution to these delivery challenges. They inherently protect cargo molecules (e.g., miRNAs) from degradation, exhibit low immunogenicity, possess intrinsic homing capacity toward injured tissues, and critically, traverse the BBB (Liu et al., 2024). Recent advances in exosome engineering further enhance their delivery potential (Nouri et al., 2024). Exosome-mediated miRNA delivery thus represents a transformative strategy to overcome limitations of conventional NP treatments through precision modulation of multiple pathological mechanisms. This review focuses on the emerging paradigm of exosome-mediated miRNA delivery for NP management. We outline the therapeutic rationale for miRNAs in NP pathogenesis and exosomes’ unique advantages as delivery vehicles, evaluate preclinical and clinical evidence for exosomal miRNA efficacy, and discuss clinical translation challenges and future directions.

## 2 Methods

This narrative review synthesizes literature retrieved from electronic databases including PubMed and ClinicalTrials.gov using core search terms: “exosomes,” “microRNA,” “neuropathic pain,” and “miRNA pain therapy” with their English equivalents. Included studies fulfilled these criteria: (1) mechanistic validation in animal or cellular models, and (2) documented evidence of exosome-mediated miRNA delivery. Exclusion criteria comprised case reports and non-English publications.

### 2.1 miRNAs in NP pathogenesis

#### 2.1.1 Expression profiles and therapeutic potential

MicroRNAs (miRNAs) are endogenous small non-coding RNAs (approximately 22 nucleotides) that regulate gene expression by binding to the 3′ untranslated region (3′ UTR) of target mRNAs. This interaction, primarily mediated by a 6–8 nucleotide seed sequence, induces mRNA degradation or translational repression (Liu et al., 2025; Sun et al., 2017; Yang X. et al., 2023). Through this post-transcriptional regulation, miRNAs orchestrate critical cellular processes in NP pathogenesis, including neuroinflammation, neuronal hyperexcitability, and impaired nerve repair (Wilkerson et al., 2020). miRNA-mRNA network analysis identified multiple dysregulated miRNAs (e.g., miR-30c-5p, miR-16-5p) and their target genes (Rnase4, Egr2), revealing inflammation-associated regulatory mechanisms in NP (Cai et al., 2020). These findings support their potential as diagnostic biomarkers and therapeutic targets. Widespread miRNA alterations occur at key pain-processing sites [dorsal root ganglia (DRG) and spinal cord] across NP models [spared nerve injury (SNI), spinal nerve ligation (SNL), chronic constriction injury (CCI), and diabetic neuropathy]. Specific miRNAs including miR-21, miR-124, and miR-146a demonstrate significant dysregulation following nerve injury (Zhong et al., 2019; Zhang et al., 2019; Lv et al., 2017). Consistent with these observations, clinical studies report abnormal expression of miR-21, miR-146a, and miR-155 in sural nerves, skin biopsies, and circulating leukocytes from patients with painful peripheral neuropathy (Leinders et al., 2017). Mechanistically, these dysregulated miRNAs converge on three core pathways: neuroinflammatory signaling modulation (TLR4/NF-κB, NLRP3 inflammasome, cytokine release via TRAF6/IRAK1/STAT3 targeting), ion channel regulation (Nav1.7, TRPV1, Kv channels), and mediation of neuroimmune interactions and neural repair processes (Yan et al., 2017; Sun et al., 2021).

The extensive dysregulation of miRNAs establishes them as potent therapeutic targets for NP, offering three primary advantages:

1. Multi-target potential: Individual miRNAs regulate gene networks governing NP mechanisms (e.g., neuroinflammation, excitability), surpassing single-target drugs.
2. Mechanism-driven efficacy: Functional restoration of specific miRNAs (e.g., miR-146a-5p) using agomirs/antagomirs alleviates pain hypersensitivity and neuroinflammation in preclinical models (Wang et al., 2018).
3. Diagnostic and predictive biomarkers: Distinct miRNA expression patterns in biofluids or tissues (e.g., elevated serum hsa-miR-19a-3p and hsa-miR-19b-3p) enable NP subtype stratification, informing personalized therapies (Tavares-Ferreira et al., 2019).

#### 2.1.2 Core regulatory mechanisms

##### 2.1.2.1 Regulation of inflammatory cascades

Within NP pathology, miRNAs exert precise control over neuroimmune interactions by targeting critical signaling nodes: During inflammation initiation, Toll-like receptor (TLR) family members including TLR4 recognize damage-associated molecular patterns (DAMPs), activating the IRAK1/TRAF6 complex through MyD88-dependent pathways. This drives NF-κB/MAPK activation and progressive release of pro-inflammatory cytokines including TNF-*α* and IL-1β (Zarezadeh Mehrabadi et al., 2022). During inflammation amplification, miR-23a inhibits IL-1β maturation in microglia by dual-targeting CXCR4 (immune cell chemokine receptor) and TXNIP (key NLRP3 activation factor) (Pan et al., 2018). Concurrently, the CXCL12/CXCR4 axis recruits immune cells such as macrophages to infiltrate injury sites, exacerbating neuroinflammation (Liu et al., 2019), whereas miR-144 and miR-140 suppress this pathway (Li et al., 2021; Zhang et al., 2020a). Through negative feedback regulation, miR-146a-5p binds the 3′ UTR of TRAF6/IRAK1 mRNAs, establishing self-limiting control of NF-κB activation (Hou et al., 2021). Additionally, miRNAs modulate inflammatory cascades by targeting transcription factors: miR-136 and miR-128-3p inhibit ZEB1, blocking pro-inflammatory gene transcription (Bao et al., 2018; Shen et al., 2019; Yan et al., 2018; Zhang et al., 2020b). miR-363-5p targets SERPING1 (regulated by SP5), conferring dual analgesic/anti-inflammatory effects that SERPING1 overexpression negates (Wu et al., 2025). Collectively, miRNAs orchestrate multi-layered regulation of neuroinflammation, establishing them as potential therapeutic targets for NP.

##### 2.1.2.2 Regulation of ion channel homeostasis

Neuronal hyperexcitability constitutes a core feature of NP, primarily driven by dysfunctional ion channel expression and activity. Exosome-mediated miRNA delivery enables novel therapeutic strategies for targeting channelopathies in voltage-gated sodium (Nav), potassium (Kv), calcium (Cav), and transient receptor potential (TRP) channels. Abnormal Nav isoform activation including Nav1.3, Nav1.7, and Nav1.8 can be selectively regulated through miRNAs: miR-30b directly targets SCN3A mRNA (Su et al., 2017); miR-182 inhibits SCN9A translation (Cai et al., 2018); and miR-7a downregulates β2 subunit (SCN2B) expression (Sakai et al., 2013), collectively reducing ectopic discharges in DRG. In contrast, miR-3584-5p exacerbates chronic constriction injury pain through Nav1.8 current suppression (Yang R. et al., 2023). Voltage-gated potassium channels critically control neuronal excitability by governing action potential generation, firing frequency, and neurotransmitter release (Manville et al., 2018; Kim and Nimigean, 2016). The miR-17-92 cluster maintains mechanical hypersensitivity post-injury through coordinated Kv regulation. This cluster contains six members—miR-17, miR-18a, miR-19a, miR-20a, miR-19b, and miR-92a-that remain persistently upregulated in injured sensory neurons (Sakai et al., 2017). Dysregulation mechanisms include miR-19a-mediated Kv4.2/4.3 mRNA targeting that reduces A-type potassium currents, and miR-137-induced Kcna2 inhibition that decreases Kv1.2 expression, both elevating neuronal excitability and pain perception. miR-137 inhibition restores Kv1.2 expression, normalizes neuronal excitability, and alleviates pain (Zhang et al., 2021). In calcium channel regulation, miR-103 targets CACNB1/CACNA2D1 (Cav1.2 auxiliary subunits) (Favereaux et al., 2011) while miR-32-5p silences Cav3.2 through histone methylation (Qi et al., 2022), both reducing calcium influx to block nociceptive sensitization. TRP channel modulation involves miR-375 and miR-455 suppressing TRPV1 expression (Li Z. et al., 2022), whereas miR-141-5p alleviates oxaliplatin-induced NP by inhibiting TRPA1 expression, thereby reducing Ca^2+^ influx and neuronal excitability (Zhang and Chen, 2021).

##### 2.1.2.3 Neural regeneration and repair

Persistent NP following nerve injury may cause cellular damage or neuronal death in spinal cord and peripheral nerve tissues (Cohen et al., 2021). Recent studies reveal that injured peripheral neurons release endogenous neurotrophic factors including brain-derived neurotrophic factor (BDNF), neurotrophin-3 (NT-3), and nerve growth factor (NGF), promoting neuronal survival and axonal regeneration (Keefe et al., 2017; Han and Xu, 2020). The bidirectional regulatory capacity of exosomal miRNAs offers unique therapeutic value for neural repair. Excessive glial scar formation impedes axonal regeneration. miRNAs balance pro-inflammatory and repair processes by targeting glial activation states: miR-503-5p alleviates NP in type 2 diabetes mellitus (T2DM) mice through suppressing SEPT9 expression in astrocytes, while miR-204 enhances sensory functional recovery by upregulating glial cell-derived neurotrophic factor (GDNF) in microglia (Guo et al., 2024; Shen et al., 2020). Concurrently, miRNAs directly regulate neuronal regeneration: miR-155 deficiency promotes axonal regeneration through enhanced SPRR1A expression (Gaudet et al., 2016), whereas miR-135a and miR-135b counteract regenerative inhibition by suppressing Kruppel-like factor 4 (KLF4) (van Battum et al., 2018). miR-210 inhibits apoptosis via ephrin-A3 (EFNA3) to support neuronal survival (Hu et al., 2016). Notably, certain miRNAs exhibit dual regulatory properties. For example, miR-21 promotes Schwann cell-mediated axonal regeneration by inhibiting EPHA4/TIMP3 (Ning et al., 2020), yet activates the epidermal growth factor receptor (EGFR) pathway to exacerbate glial scarring (Kar et al., 2021). Across various neural injury models, miR-21 demonstrates functional versatility by modulating multiple signaling pathways to promote neural repair (Ning et al., 2020; Kar et al., 2021; Li et al., 2018).

These findings demonstrate that miRNAs therapeutically target core NP mechanisms (Table 1). However, clinical translation faces challenges including nuclease-mediated degradation and inadequate targeting specificity (Pedder et al., 2025; Wang and Wu, 2024). Developing stable delivery systems for miRNA mimics/inhibitors remains a critical unmet need (Zhang et al., 2017).

**Table 1:** Core mechanisms of miRNA targeted intervention in neuropathic pain.

Mechanism	miRNA	Model	Target	Effect	Objective	References
Neuroinflammation	miR-23a↑	pSNL	CXCR4/TXNIP/NLRP3↓	Ameliorates mechanical allodynia	C57BL/6 mice	Pan et al. (2018)
	miR-140↑	CCI	S1PR1↓	Ameliorates mechanical allodynia	SD rats	Li et al. (2021)
	miR-144↑	CCI	RASA1↓	Alleviates mechanical allodynia	C57BL/6 mice	Zhang et al. (2020a)
	miR-146a-5p↑	CCI	IRAK1/TRAF6↓	Suppresses mechanical allodynia and thermal hyperalgesia	SD rats	Hou et al. (2021)
	miR-136↑	CCI	ZEB1↓	Ameliorates mechanical allodynia	SD rats	Shen et al. (2019)
	miR-128-3p↑	CCI	ZEB1↓	Suppresses mechanical allodynia and thermal hyperalgesia	SD rats	Zhang et al. (2020b)
	miR-363-5p↑	CCI	SERPING1↓	Suppresses mechanical allodynia and thermal hyperalgesia	SD rats	Wu et al. (2025)
Neuronal ion channels	miR-30b↑	SNL	SCN3A (Nav1.3)↓	Attenuates NP	SD rats	Su et al. (2017)
	miR-182↑	SNI	SCN9A (Nav1.7)↓	Attenuates NP	SD rats	Cai et al. (2018)
	miR-7a↑	CCI/SNL	β2 subunit (SCN2B)↓	Attenuates NP	SD rats	Sakai et al. (2013)
	miR-3584-5p↑	CCI	ERK5/CREB (Nav1.8)↓	Aggravates NP; promotes apoptosis	SD rats	Yang R. et al. (2023)
	miR-17-92↓	SNL	Multiple voltage-gated K^+^ channels↑	Alleviate NP	SD rats	Sakai et al. (2017)
	miR-137↓	CCI	Kcna2 (Kv1.2)↑	Reduces tactile sensitivity; increases thermal sensitivity	SD rats	Zhang et al. (2021)
	miR-103↑	SNL	Cav1.2-LTC↓	Attenuates NP	Wistar rats	Favereaux et al. (2011)
	miR-32-5p↑	CCI-ION	Cav3.2↓	Attenuates NP	SD rats	Qi et al. (2022)
	miR-141-5p↑	Oxaliplatin (OXA)	TRPA1↓	Attenuates NP	SD rats	Zhang and Chen (2021)
Neural repair	miR-503-5p↑	DPN	SEPT9↓	Reduces astrocyte activation and ameliorates NP	db/db mice	Guo et al. (2024)
	miR-155↓	SCI	SPRR1A↑	Reduces inflammatory signaling; promotes neuronal survival and neurite growth	C57BL/6 mice	Gaudet et al. (2016)
	miR-135a/b↑	ONI	KLF4↓	Promotes axon regeneration	C57BL/6 mice	van Battum et al. (2018)
	miR-21↑	SNL	TGFβI/TIMP3/EPHA4↓	Facilitates SC proliferation and axon regeneration	SD rats	Ning et al. (2020)
	miR-21↑	ONC	EGFR↑	Facilitates axon regeneration	SD rats	Li et al. (2018)
	miR-21↑	SNI	PTEN↓	Facilitates axon regeneration	SD rats	Kar et al. (2021)

pSNL, partial sciatic nerve ligation; CCI, chronic constriction injury; SNL, spinal nerve ligation; SNI, spared nerve injury; SCI, spinal cord injury; DPN, diabetic peripheral neuropathy; ONI, optic nerve injury; CCI-ION, chronic constriction injury of infraorbital nerve; OXA, oxaliplatin (chemotherapy-induced neuropathy model); ↑, denotes miRNA overexpression intervention; ↓, indicates miRNA suppression intervention; Arrows (↑/↓), signify direction of target expression changes (e.g., NLRP3↓ = inflammasome suppression).

### 2.2 Overview of exosomes

#### 2.2.1 Biological properties

Exosomes represent a subtype of extracellular vesicles (EVs) ranging from 50–150 nm in diameter. They are distinguishable from microvesicles (100–1,000 nm) by surface markers CD63/CD9 (Meldolesi, 2018). These vesicles form through bilayer invagination of cellular membranes and are subsequently released from multivesicular bodies (MVBs) (Yang et al., 2024). Though initially considered cellular waste disposal machinery, exosomes secreted by all cell types—including immune cells and neurons—are now recognized as crucial mediators of intercellular and intracellular communication. MVBs reside within cell bodies of DRG sensory neurons, which may release EVs (including exosomes) under appropriate conditions. Exosomes contain functionally diverse proteins essential for cell adhesion, membrane fusion, metabolism, and signal transduction. Beyond proteins, they carry multiple nucleic acids including miRNAs, messenger RNAs (mRNAs), DNA fragments, and long non-coding RNAs. These constituents mediate intercellular signaling in biological processes such as immune modulation and neural transmission (Bahram Sangani et al., 2021). Notably, exosomes are widely present in bodily fluids and transmit molecular signals via paracrine, autocrine, or endocrine pathways (Chen et al., 2019). Their biogenesis occurs in virtually all cell types (Habib et al., 2023), with particularly active production observed in tumor cells (Graner, 2019), immune cells, and neural cells. Emerging evidence indicates cellular origin critically determines both exosomal cargo composition and biological functionality (Yang et al., 2025). Substantial differences in contents, surface markers, and functions exist among exosomes derived from distinct cell types, suggesting specialized roles in biological processes.

#### 2.2.2 Biogenesis and secretion processes

Though several mechanisms of exosome biosynthesis and secretion have been identified, many aspects remain incompletely understood. Exosome formation constitutes a complex multistep process involving membrane budding, invagination, multivesicular body (MVB) formation, and ultimate secretion (Ren et al., 2024). Recent studies demonstrate that exosome generation primarily relies on the intraluminal vesicle (ILV) formation pathway (Ghosh et al., 2024), comprising both ESCRT-dependent and ESCRT-independent mechanisms (Bavafa et al., 2025). The ESCRT (endosomal sorting complexes required for transport) complexes drive ILV generation through membrane remodeling and cargo sorting (Larios et al., 2020). Specifically, the ESCRT-0 complex recognizes and recruits cargo proteins, while ESCRT-I and ESCRT-II collectively promote membrane invagination, and ESCRT-III mediates vesicle fission and release (Ju et al., 2021). Precise regulation of the ESCRT machinery is critical not only for ILV formation but also for exosome secretion (Horbay et al., 2022). Beyond ESCRT-dependent pathways, exosome biogenesis involves alternative mechanisms (Yang et al., 2024). Lipid molecules including ceramide play pivotal roles by altering membrane lipid composition, enhancing fluidity, and facilitating ILV generation (Yanagawa et al., 2024). Furthermore, phosphatidylinositol 3-kinase (PI3K) and its product phosphatidylinositol-3,4,5-trisphosphate (PIP3) significantly contribute to exosome production (Yao et al., 2025). Exosome trafficking and release constitute equally complex processes governed by molecular regulators such as Rab GTPases (e.g., Rab27a/b) (Kim et al., 2024). These GTPases regulate MVB-plasma membrane fusion to ensure precise exosome transport and secretion. Recipient cells internalize exosomes primarily through endocytosis, direct membrane fusion, or surface receptor interactions (Vučemilović, 2024). Though endocytosis represents the predominant mechanism, direct fusion offers greater therapeutic promise for drug delivery due to enhanced intracellular cargo release efficiency (Hushmandi et al., 2024). These discoveries deepen our understanding of exosome biogenesis while establishing theoretical foundations for novel therapeutic strategies.

### 2.3 Advantages of exosomes as delivery vehicles

#### 2.3.1 Natural targeting capacity

The targeting capacity of exosomes primarily depends on their surface characteristics and molecular cargo. Research confirms that multiple specific proteins on exosomal surfaces mediate interactions with target cells. Notably, lysosome-associated membrane glycoprotein 2B (LAMP2B) enhances exosomal binding to neurons and their subsequent internalization, establishing its role as a key targeting protein (Qiao et al., 2023). This property provides natural targeting advantages for exosomal drug delivery. TGF-β1-induced human umbilical cord smooth muscle cell (hUCSMC)-derived exosomes exhibit effective targeting toward microglia, suppressing microglial hyperplasia and alleviating NP. Mechanistic studies reveal that urothelial cancer-associated 1 (UCA1) directly interacts with miR-95-5p to release FOXO3a expression (Mou et al., 2023). These natural targeting mechanisms enhance therapeutic efficacy while reducing impacts on non-target cells and minimizing potential side effects. Additionally, exosomes support targeting through neuronal communication functions. By binding directly to neurons and modulating their physiological states, exosomes influence pain perception and processing (Frühbeis et al., 2013). Following peripheral axonal injury, DRG sensory neurons release exosomes enriched with upregulated miR-21-5p. These exosomes are readily phagocytosed by macrophages, promoting pro-inflammatory polarization and inflammatory factor release. Intrathecal administration of miR-21 inhibitors prevents macrophage infiltration and NP development (Simeoli et al., 2017). Thus, tissue-specific exosome delivery circumvents detrimental neuron-macrophage communication, offering novel therapeutic opportunities for NP.

#### 2.3.2 Ability to cross the blood-brain barrier

BBB comprises tight junctions between endothelial cells in brain capillaries, primarily protecting the CNS from harmful substances (Lerussi et al., 2025). However, this barrier also restricts entry of many therapeutics, complicating neurological disorder treatment (Gong et al., 2025). Consequently, identifying carriers capable of crossing the BBB has become a research priority. Exosomes emerge as ideal candidates due to their natural biocompatibility and low immunogenicity. Exosomes can traverse the BBB bidirectionally between bloodstream and brain, though specific mechanisms for peripheral-to-brain migration remain incompletely elucidated (Banks et al., 2020). Exosomes primarily cross the BBB via transcytosis-transporting through intracellular compartments similarly to immune cells and pathogens-rather than paracellular routes through extracellular spaces. Post-crossing, two functional possibilities exist: complete traversal of the endothelial barrier for global brain effects (Khan et al., 2022), or sequestration within brain endothelial cells influencing these cells and triggering specific transport mechanisms (Saeedi et al., 2019; Console et al., 2019). This transmigration capability extends beyond neural stem cell (NSC)-derived exosomes. Other exosomes, including those from bone marrow mesenchymal stem cells (MSCs) and placental tissue, demonstrate similar BBB-crossing capacities. Clinically, exosomes’ penetrative ability positions them as novel neurological therapeutics. MSC-derived exosomes alleviate NP in chronic models by suppressing microglial activation and reducing neuroinflammation (Gao et al., 2023b). Moreover, exosomes show potential for delivering therapeutic molecules including miRNAs and proteins that modulate pain-processing and inflammatory pathways (Kang and Guo, 2022; Di Ianni et al., 2025).

#### 2.3.3 Low immunogenicity and high stability

Exosomal biocompatibility enables prolonged systemic circulation without immune recognition or clearance. This property permits effective therapeutic molecule delivery, enhancing efficacy while minimizing side effects. The membrane structure protects encapsulated bioactive components, maintaining stability *in vivo* and *in vitro* (Tang et al., 2021). Studies confirm prolonged exosomal circulation effectively avoids clearance by the reticuloendothelial system (Patil et al., 2020). Compared to traditional drug delivery systems, exosomes better preserve therapeutic activity and achieve higher concentrations in target tissues. Notably, surface molecules including CD47, CD24, CD44, and CD31 function as anti-phagocytic signals, helping exosomes evade phagocytic clearance by macrophages. This enhances systemic stability and bioavailability (Parada et al., 2021). As naturally derived carriers, exosomes show minimal long-term accumulation in organs compared to viral vectors, resulting in negligible systemic toxicity (Yang et al., 2015). Recent research reveals exosomes provide protection during thermal stress by transferring thermotolerance signals that help cells maintain viability under extreme conditions (Logan et al., 2024).

Exosomes constitute highly efficient miRNA delivery vehicles due to their biocompatibility, low immunogenicity, and rapid membrane fusion capacity (Bian et al., 2025). To systematically compare advantages, Table 2 details exosome-based delivery versus conventional therapies across targeting specificity, blood-brain barrier penetration, and side effects.

**Table 2:** Comparison of exosome-based delivery systems vs. conventional therapies.

Comparison criteria	Exosome-based delivery systems	Conventional therapies (e.g., opioids, anticonvulsants)	References
Targeting specificity	Achieves tissue/cell-specific delivery via surface modifications (e.g., CD47, antibodies), minimizing off-target effects	Non-specific systemic distribution, relying on passive diffusion driven by physicochemical properties (e.g., lipophilicity)	Parada et al. (2021) and Yang et al. (2015)
Blood-brain barrier penetration	Naturally excels in crossing the BBB or via intranasal administration for direct CNS delivery	Limited penetration for most drugs, requiring high doses with increased side effects	Zhao et al. (2025) and Zhou et al. (2023)
Side effects	Low immunogenicity (autologous sources), no risk of addiction or respiratory depression	High side-effect burden (e.g., opioid addiction, anticonvulsant-induced sedation)	Arthur et al. (2025) and Leão Nunes Filho et al. (2024)
Immunomodulatory effects	Carries anti-inflammatory miRNAs to suppress microglial activation and synergistically alleviate neuroinflammation	Lacks direct immunomodulatory function; may exacerbate inflammation (e.g., chronic opioid use)	Kaye et al. (2024) and Hua et al. (2022)
miRNA regulatory network	Capable of delivering multiple miRNAs to synergistically suppress inflammation and ion channel activation	Single-target action, unable to modulate complex regulatory networks	Lv et al. (2017) and Su et al. (2017)

### 2.4 Exosome-mediated miRNA therapy: evidence and mechanisms

#### 2.4.1 Preclinical model evidence: validation of efficacy and mechanism

##### 2.4.1.1 Exosome-delivered miRNA targeting neuroinflammation: mechanisms underlying analgesia

Exosomal miRNAs exhibit high stability and amplification potential due to their lipid bilayer structure, enabling traversal across blood-brain or blood-spinal cord barriers to mediate analgesia in chronic pain models (Huh et al., 2017; Ding et al., 2019). In CCI rat models, miR-181c-5p expression is significantly downregulated, while intrathecal delivery of exosomal miR-181c-5p alleviates NP and neuroinflammatory responses (Zhang et al., 2022). Exosomal miRNAs operate through autocrine secretion and transport to target sites, acting on macrophages, microglia, neurons, or other tissue cells to regulate inflammatory factor secretion and oxidative stress, thereby modulating NP pathogenesis. In diabetic nephropathy (DN) mouse models, macrophage-derived EVs enriched with miR-21-5p enhance pyroptosis by upregulating A20 (a negative regulator of the NF-κB pathway). Correspondingly, intrathecal administration of anti-miR-21-5p antibodies reduces dorsal root ganglion DRG hyperalgesia and macrophage recruitment (Ding et al., 2021). Similarly, human umbilical cord mesenchymal stem cell (huc-MSC)-derived exosomes regulate microglial pyroptosis and autophagy through the miR-146a-5p/TRAF6 axis (Hua et al., 2022). Certain exosomal miRNAs alleviate neuroinflammation by inhibiting pro-inflammatory cytokine production or promoting anti-inflammatory cytokine release. For instance, human umbilical cord MSC-derived exosomes upregulate autophagy proteins LC3-II and beclin1 while blocking NLRP3 inflammasome activation via miR-146a-5p/TRAF6 signaling in the spinal dorsal horn (Hua et al., 2022). Bone marrow mesenchymal stem cell-derived extracellular vesicles (BMSC-EVs) enriched with miR-23b regulate TLR4/NF-κB signaling, attenuating inflammation and improving pathological status in SCI rats (Nie and Jiang, 2021). Additionally, BMSC-derived exosomes promote miR-145-5p expression to inhibit TLR4/NF-κB pathway activation, demonstrating significant anti-inflammatory and pathway regulatory effects in both SCI rats and PC12 cells (Jiang and Zhang, 2021). These findings highlight the therapeutic value of exosomal miRNAs in controlling neuroinflammatory signaling and ameliorating neural damage.

##### 2.4.1.2 Synergistic protective mechanism of exosome-mediated miRNA in nerve regeneration and anti-apoptosis

**Figure 1:**
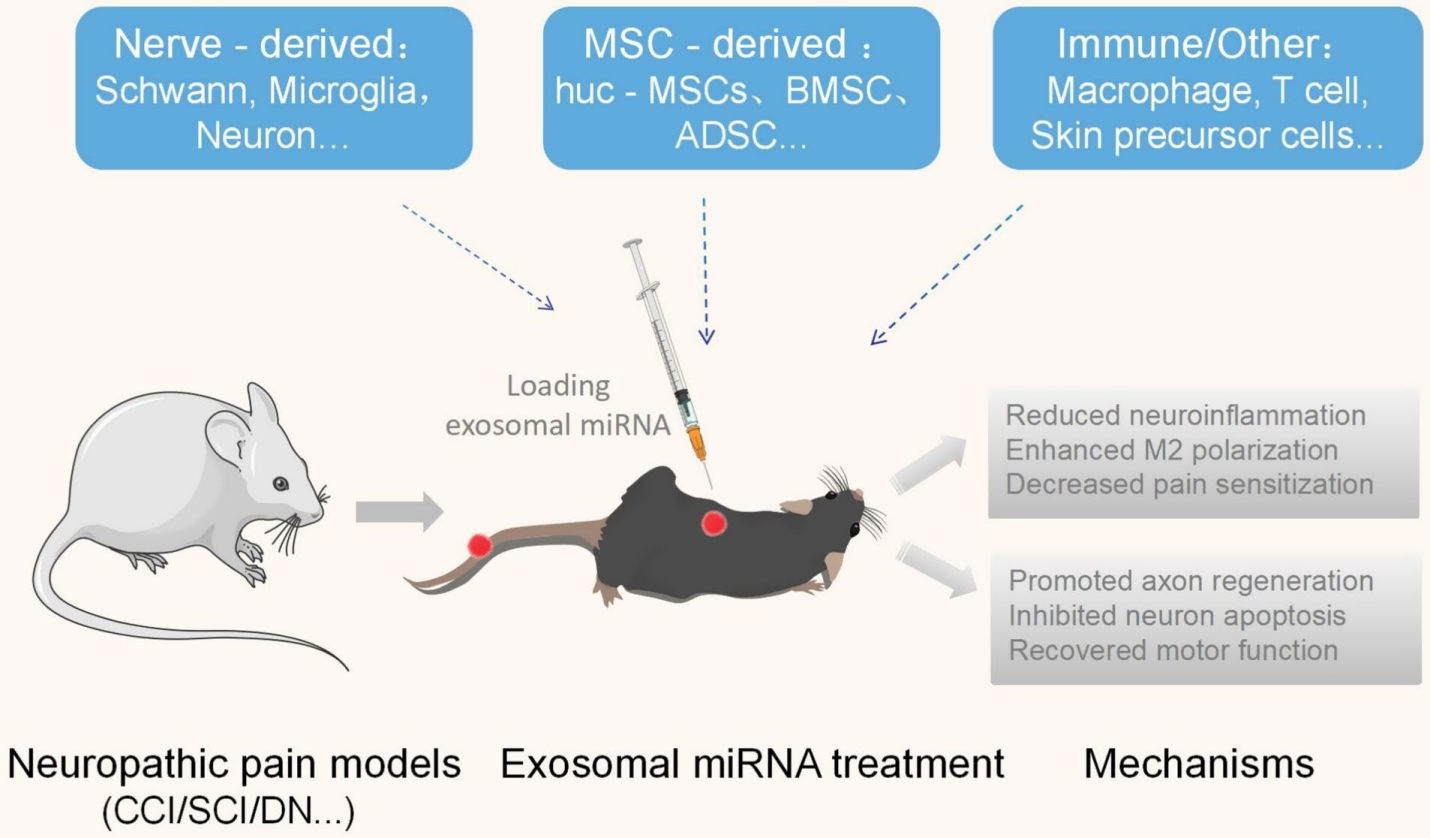
Exosomal miRNA treatment for neuropathic pain. Schematic illustration of exosomal miRNA-based therapeutic strategies for neuropathic pain. Neuropathic pain models (e.g., CCI, SCI, DN) are subjected to exosomal miRNAs sourced from multiple cellular origins: nerve-resident cells (Schwann cells, microglia, neurons), mesenchymal stem cells (huc-MSCs, BMSCs, ADSCs), and immune/other lineages (macrophages, T cells, skin precursor cells). These exosomal miRNAs modulate neuropathic pain pathophysiology through dual-pronged mechanisms: ① anti-inflammatory actions (reducing neuroinflammation, promoting M2 polarization, decreasing pain sensitization); ② neuroregenerative effects (enhancing axon regeneration, suppressing neuron apoptosis, restoring motor function).

Exosomes derived from Schwann cells, macrophages, and mesenchymal stem cells (MSCs) promote peripheral nerve regeneration (Sanchez et al., 2017; Marofi et al., 2017; Mead and Tomarev, 2017). Studies confirm exosomes facilitate regeneration of damaged nerves and improve motor function recovery in regenerated nerves in rat sciatic nerve compression models (Bucan et al., 2019). Exosomes enriched with the miR-17-92 cluster activate the PI3K/Akt/mTOR/GSK-3β signaling pathway by targeting PTEN, increasing neural plasticity and functional recovery (Xin et al., 2017). Specifically, skin precursor-derived Schwann cell extracellular vesicles (SKP-SC-EVs) containing miR-21-5p enhance DRG sensory neuron growth and survival through the PTEN-PI3K pathway (Cong et al., 2021). Simultaneously, miR-23b-3p promotes axonal regeneration by directly targeting Nrf1 mRNA (Xia et al., 2020). Regarding SCI models, intrathecal injection of MSC-derived exosomes significantly upregulates miR-99b-3p expression while activating microglial autophagy and alleviating mechanically induced allodynia caused by microglial activation (Gao et al., 2023a). Concurrently, exosomes from miR-126-modified MSCs reduce neuronal apoptosis while promoting functional regeneration (Huang et al., 2020), and exosomes derived from both MSCs and human neuroepithelial stem cells—enriched with miR-29b—downregulate PTEN and caspase-3 to inhibit neuronal apoptosis and confer therapeutic efficacy for SCI (Yu et al., 2019). Exosomal miR-499a-5p plays a neuroprotective role in SCI by targeting the JNK3/c-jun apoptotic pathway, reducing nerve cell apoptosis post-injury while decreasing cavity formation in lesioned areas. This mechanism promotes functional hindlimb recovery in rats through adipose-derived mesenchymal stem cell exosomes (ADSC-EXs) carrying miR-499a-5p, which reduce JNK3 expression and diminish nerve cell death after SCI (Liang et al., 2022). Adipose-derived mesenchymal stem cells (ADSCs) contain abundant neurotrophic factors, immunomodulatory factors, and angiogenic factors associated with neuronal differentiation and nerve regeneration (Harrell et al., 2022). Through miRNA transport, exosomes enhance neuronal cell activity and reduce apoptosis during early stages, thereby promoting functional recovery (see Figure 1).

Thus, exosome-mediated miRNA transfer represents an effective therapeutic approach for NP (Table 3).

**Table 3:** Exosomal miRNAs in preclinical models of neuropathic pain: therapeutic efficacy and mechanisms.

miRNA	Model	Exosome source	Delivery method	Target	Effect	Study type	Objective	References
miR-146a-5p↑	CIP	huc-MSCs	Intrathecal injection	TRAF6/NLRP3↓	Mechanical allodynia and thermal hyperalgesia; reduces neuroinflammation	*In vitro* + *in vivo*	C57BL/6 mice	Hua et al. (2022)
miR-181c-5p↑	CCI	BMSC-EVs	Intrathecal injection	IL-6/IL-1β/TNF-α↓	Attenuates NP and neuroinflammation	*In vivo* + *in vitro*	SD rats	Zhang et al. (2022)
miR-21-5p↓	DN	Macrophage-derived	Tail vein injection	A20/NF-κB↓	Attenuates NP	*In vivo* + *in vitro*	C57BL/6 mice	Ding et al. (2021)
miR-23b↑	SCI	BMSC-EVs	Tail vein injection	TLR4/NF-κB↓	Reduces inflammation; improves spinal injury recovery	*In vitro* + *in vivo*	SD rats	Nie and Jiang (2021)
miR-21-5p↑	PNI	SKP-SCs	*In vitro* treatment	PTEN/PI3K↓	Improves neurite growth in DRG sensory neurons	*In vitro*	SD rats	Cong et al. (2021)
miR-99b-3p↑	CCI	hUC-MSCs	Intrathecal injection	PI3K/AKT/mTOR↓	Promotes autophagy; alleviates pain	*In vivo* + *in vitro*	SD rats	Gao et al. (2023a)
miR-126↑	SCI	huc-MSCs	Tail vein injection	SPRED1/PIK3R2↓	Promotes neurogenesis; alleviates pain	*In vivo* + *in vitro*	SD rats	Huang et al. (2020)
miR-29b↑	SCI	BMSCs	Tail vein injection	NF200/GAP-43↑	Promotes neural regeneration; alleviates pain	*In vivo* + *in vitro*	SD rats	Yu et al. (2019)
miR-499a-5p↑	SCI	ADSCs	Tail vein injection	JNK3/c-jun↓	Reduces neuronal apoptosis; improves motor function recovery	*In vivo* + *in vitro*	SD rats	Liang et al. (2022)

BMSC, bone marrow mesenchymal stem cells; ADSC, adipose derived stem cells; hucMSC, human umbilical cord mesenchymal stem cells; SKPSCs, skin derived precursor Schwann cells; CCI, chronic constriction injury; SCI, spinal cord injury; CIP, chemotherapy induced peripheral neuropathy; DN, diabetic neuropathy; PNI, peripheral nerve injury.

#### 2.4.2 Clinical investigations

We identified clinical trials evaluating exosomes as therapeutic agents for NP (Table 4), including one published clinical trial and one ongoing registered trial (data current through June 2025). These investigations provide foundational insights into the complex mechanistic actions of exosomal miRNAs.

**Table 4:** Clinical trials of exosome-based therapies for neuropathic pain.

Trial ID	Condition	Participants	Exosome source	Study design	Intervention	Phase	Status	Primary Findings	Limitations
IRCT20200502047277N1	SCI	9	HUC-MSCs	Non-randomized/open label	Intrathecal injection(Single dose: 300 μg)	I	Completed	Sensory improvement in 4/9 patients (ΔASIA Sensory Score ↑); no early/late AEs	Invasive delivery; small sample (*n* = 9); single-center
NCT05152368	Peripheral neuropathy/trigeminal neuralgia	20	UC-MSCs	Non-randomized/openlabel	Intranasal instillation (single dose: 8 × 10^10^ particles)	I	Recruiting (Est. completion: January 2026)	Non-invasive BBB bypass; Ongoing safety monitoring	Results pending

AEs, adverse events; ASIA, American Spinal Injury Association; ΔASIA↑: exceeded minimal clinically important difference (MCID). Dose: 8 × 10^10^ particles = 800B exosomes (MISEV-compliant).

The completed Phase I trial IRCT20200502047277N1 (Akhlaghpasand et al., 2024) adopted a single-center design focused on safety assessment rather than efficacy evaluation. Although preliminary improvements demonstrate clinical relevance, natural disease progression complicates definitive efficacy determination. Observed sensory-motor functional enhancements in certain patients may derive from either exosomal therapeutic effects or spontaneous disease resolution. This reflects methodological constraints in current efficacy evaluation approaches, necessitating larger multicenter randomized Phase II/III trials to validate outcomes and establish the definitive therapeutic role of exosome therapy in NP. Safety monitoring revealed no severe adverse events. However, long-term biological consequences of exosomes *in vivo* remain undetermined. Potential concerns include sustained biological activity, immune response induction, and interference with normal cellular functions. Notably, engineered exosomal products warrant particularly rigorous risk assessment due to greater uncertainty.

Regrettably, current clinical data demonstrate: limited study accessibility, data incompleteness (e.g., pending NCT05152368 results), and recurrent methodological limitations: small cohorts, abbreviated follow-up periods, and non-blinded designs lacking placebo controls. These constraints impede comprehensive scientific assessment.

### 2.5 Evolving paradigms in exosome delivery: administration routes and biomaterial innovations

#### 2.5.1 Comparative analysis of exosome delivery routes

Delivery routes critically influence therapeutic efficacy due to their significant impacts on exosome distribution, absorption, and functional outcomes. Different administration methods—including intrathecal injection and intranasal instillation—distinctly affect exosome biodynamics. Intrathecal injection achieves targeted delivery to injury sites, maximizing local effects while minimizing systemic side effects. Epidural injection specifically targets spinal cord tissue with reduced complication rates. Intravenous administration remains the predominant preclinical delivery route (Hassanzadeh et al., 2021), offering systemic distribution with technical simplicity and lower complication risks via caudal vein injection.

Two registered clinical studies utilize intrathecal injection and intranasal instillation. Intranasal administration leverages olfactory and trigeminal axonal pathways to bypass the blood–brain barrier, enabling direct therapeutic delivery to brain tissue. Compared to invasive approaches (intrathecal or parenchymal delivery) with infection risks, this non-invasive technique offers significant advantages. Multiple preclinical studies confirm intranasally administered exosomes effectively prevent neuronal apoptosis and improve neurological recovery (Gotoh et al., 2025).

#### 2.5.2 Engineering strategies for enhanced targeting

Exosomes exhibit unique advantages as natural drug carriers, but their clinical translation is limited by insufficient targeting specificity. For example, intravenous administration leads to rapid clearance by phagocytic organs such as the liver and spleen, significantly reducing target organ enrichment efficiency. This not only diminishes therapeutic efficacy but also raises risks of off-target toxicity. Novel exosome engineering techniques enhance delivery precision through customized miRNA loading and surface modifications (Del Pozo-Acebo et al., 2021). Research has developed genetically engineered exosomes carrying miR-21 combined with collagen-I (Col-I) scaffolds to repair SCI, demonstrating improved stability, delivery efficiency, and targeting (Liu et al., 2022).

Additionally, researchers encapsulated adipose tissue-derived mesenchymal stem cell exosomes (AD-MSC-EXs) within collagen and fibrin hydrogels (Afsartala et al., 2023), extending active retention at injury sites in SCI rat models. Gelatin sponge (Gelfoam)-loaded human umbilical cord mesenchymal stem cell exosomes (HucMSC-EXs) achieve precise delivery to SCI sites while promoting neural regeneration (Poongodi et al., 2024). Innovative tetrahedral DNA nanostructure (TDN)-based delivery systems incorporate RNase H-sensitive DNA–RNA hybrid sequences as bioswitches. Upon reaching target cells (e.g., in inflammatory or tumor microenvironments), RNase H specifically cleaves hybrid strands to trigger precise miRNA release (Li et al., 2025).

These engineering strategies can overcome biological barriers including phagocytic clearance and short half-life through surface functionalization, hydrogel sustained-release systems, and responsive nanoswitch designs, significantly enhancing spatiotemporal delivery precision.

### 2.6 Clinical translation: potential and challenges

#### 2.6.1 Key challenges

How to address miRNA target gene complexity and functional validation bottlenecks?

How to predict exosomal miRNA therapeutic efficacy in individual patients?

What defines long-term *in vivo* distribution and safety profiles of therapeutic exosomes?

How to establish GMP-compliant large-scale exosome production?

How to optimize storage conditions to improve clinical feasibility?

How to reduce exosomal immunogenicity to enhance biological safety?

#### 2.6.2 Complexity of miRNA target genes and challenges in functional validation

Neuropathic pain (NP) pathogenesis involves diverse cellular and molecular mechanisms. Different NP subtypes-including chemotherapy-induced, diabetic, and traumatic NP-exhibit distinct miRNA expression profiles and functional pathways. NP models demonstrate 2,776 differentially expressed RNA molecules comprising 219 miRNAs and 2,557 mRNAs. Crucially, miRNAs regulate multiple target genes simultaneously, frequently through indirect mechanisms, significantly complicating target identification (Li et al., 2019; Golmakani et al., 2024). Current bioinformatics tools predict potential targets but lack experimental validation, undermining prediction reliability. These regulatory interactions are further complicated by cell-type specificity, microenvironmental influences, and competitive binding with non-coding RNAs (e.g., lncRNAs and circRNAs) (Mukherjee et al., 2025). miRNA regulatory networks exhibit dynamic complexity, with target specificity varying across physiological and pathological states. For instance, while miR-133a-3p overexpression attenuates microglial activation and neuroinflammation in CCI models (Jia et al., 2021; Gao et al., 2024), its upregulation conversely promotes neuroinflammation and pain development in diabetic NP (DNP) models through TRAF6 and PIAS3 protein modulation (Chang et al., 2020). Accurately interpreting miRNA functions in neuropathology requires integrated analysis of both multi-target characteristics and cell-contextual functionality. Emerging technologies-particularly single-cell RNA sequencing, NGS, and machine learning algorithms (Picchio et al., 2025)-will likely advance miRNA-target identification, accelerating NP therapeutic development.

#### 2.6.3 Scale-up: preparation, purification and quality control challenges

Exosome isolation employs diverse methods including ultracentrifugation, ultrafiltration, polymer precipitation, and immunoaffinity techniques. However, while ultracentrifugation remains the most common isolation technology, it demands specialized equipment and technical expertise while often causing damage to exosomes and functional loss (Baruah et al., 2024). Furthermore, exosome isolation is frequently contaminated by coexisting extracellular vesicles (such as microvesicles and apoptotic bodies), complicating purification processes and compromising analytical accuracy (Marjani et al., 2024). Exosomes contain diverse components including proteins, lipids, and RNAs, with biological activity closely linked to compositional integrity. Therefore, ensuring quality during preparation—particularly evaluating purity and bioactivity—represents an urgent challenge (Zhang F. et al., 2024). Currently, no standardized criteria exist to assess exosomal quality, hindering clinical translation (Sen et al., 2023; Fan et al., 2024). The International Society for Extracellular Vesicles (ISEV) aims to address these issues through its 2023 guidelines, providing technical guidance for documenting specific functional activities and procedural steps (Théry et al., 2018). Researchers are developing new technologies and standards: Microfluidics-based approaches enable improved isolation efficiency with reduced exosomal damage (Xing et al., 2025), while combined high-throughput analytical techniques and biological functional testing allow more comprehensive evaluation of exosomal quality and bioactivity (Zhang J. et al., 2024; Ishii and Tateno, 2025). These measures will enhance production consistency while ensuring clinical safety and efficacy. Future research should focus on developing efficient isolation technologies and rigorous quality control systems to achieve clinical translation of exosome-based therapies.

#### 2.6.4 Stability and biosafety of delivery systems

Delivery system stability critically determines therapeutic duration and effects *in vivo*. Multiple studies demonstrate that exosomes exhibit optimal stability when stored at −80°C (Levy et al., 2023), though this condition proves impractical for routine clinical application. Optimizing storage conditions is essential to ensure reliability and effectiveness in clinical settings. Current research lacks sufficient data regarding the shelf life and *in vivo* stability of exosomal preparations (Palakurthi et al., 2024), limiting their clinical translation. Substantial technical challenges remain unresolved, including controversial issues surrounding administration routes, injection rates, and dosage standardization (Wang et al., 2023; Batrakova and Kim, 2015). Notably, *in vivo* studies show no significant positive correlation between exosome dosage levels and neuroregenerative outcomes, with higher doses failing to enhance therapeutic efficacy (Zhao et al., 2020). Certain delivery systems may activate immune responses, inducing inflammation or other adverse effects that compromise treatment effectiveness (Brain et al., 2021). Some researchers have utilized cell-targeted delivery systems (CDSEMs) constructed from edible materials (Li X. et al., 2022) and delivery systems fabricated using polymer matrices and other materials, which demonstrate outstanding performance in maintaining miRNA bioactivity and reducing immune responses *in vivo*, significantly enhancing biosafety (Poongodi et al., 2024). The biosafety of exosomes is significantly influenced by their cellular origin and purification processes. While exosomes derived from healthy cells demonstrate favorable safety profiles *in vivo*, those originating from pathological conditions may cause adverse reactions (Sohrabi et al., 2022; Yamayoshi et al., 2020). Comprehensive assessment of *in vivo* immune responses and potential side effects is mandatory before clinical application. Strict compliance with GMP (good manufacturing practice) standards during manufacturing processes is essential to mitigate patient risks. Metabolomics can identify off-target effects and adverse drug events by detecting early signs of drug-induced liver injury, cardiotoxicity, and other complications through metabolic profile analysis (Shaman, 2024; Røikjer et al., 2024). The incomplete functional characterization of exosomes hinders accurate prediction of their long-term safety and efficacy. Although surface modification with targeting peptides significantly enhances exosomal targeting capability, potential immunogenicity of these peptides raises concerns about immune responses in humans. Developing secure and effective methods for anchoring targeting peptides to exosomes thus remains a challenging pursuit.

## 3 Future directions

**Figure 2:**
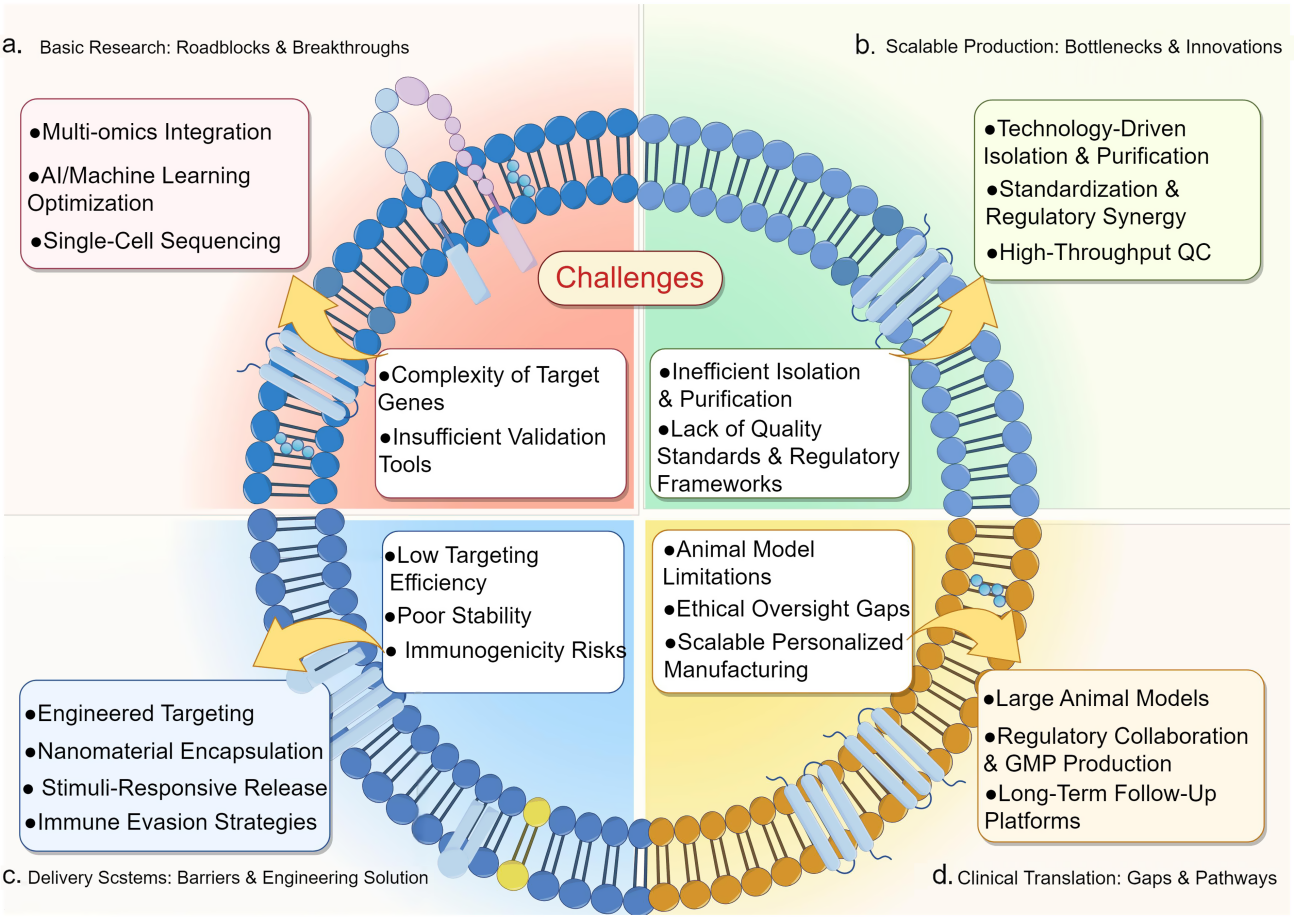
Exosome-mediated miRNA delivery for neuropathic pain: design challenges and clinical pathways. Schematic of exosome design for neuropathic pain therapy, with challenges as the core. It maps hurdles across target gene complexity, production inefficiencies, delivery barriers, and clinical translation gaps to solutions (multi-omics, engineered targeting, regulatory pathways), illustrating how overcoming these accelerates exosome-mediated miRNA delivery to reshape NP treatment outcomes.

1. Integrating multi-omics technologies—including transcriptomics, proteomics, and metabolomics—will enable systematic elucidation exosomal miRNA mechanisms in NP. Researchers integrating genome-wide association studies (GWAS) with multi-omics data have revealed significant overlap in gene co-expression modules between NP and inflammatory pain (IP). Furthermore, integrated multi-omics analyses have identified specific miRNAs critically regulating neuroinflammation and neuronal excitability, while uncovering novel miRNA targets and signaling pathways (Ye et al., 2022). Leveraging omics technologies to select optimal donors and optimize exosome composition may consequently improve therapeutic outcomes (Lotfy et al., 2023).
2. Engineering exosomes through surface modifications (aptamers, antibodies, peptides) can enhance targeting precision to injured spinal cord regions and specific cell types, improving both delivery accuracy and therapeutic efficacy (Ye et al., 2023). Integrating exosomes with nanomedicine, materials science, and bioengineering could augment their therapeutic potential as delivery vehicles (Haroon et al., 2024). For instance, the RNAi-Tim3-Exo@SF hydrogel system delivers siRNA-Tim3-modified exosomes to precisely regulate Tim3 expression. This system stabilizes microtubules, promotes axonal regeneration, stimulates angiogenesis, modulates inflammatory microenvironments, and significantly improves motor function in spinal cord injury models. The key reparative mechanisms likely involve miR-155-5p within RNAi-Tim3-Exo (Dong et al., 2025). Such integrated strategies combining immunomodulation with tissue engineering may represent effective approaches for future clinical applications.
3. Current NP research minimally addresses large-animal models (pigs, non-human primates) in the literature. These species demonstrate greater neurological similarity to humans in axonal diameter, myelination patterns, and glial responses, enabling superior modeling of human pathological changes and pain behavior following neural injury (Karri et al., 2022). Consequently, the field urgently requires transitioning from exclusive rodent models to incorporating large-animal paradigms (e.g., porcine sciatic nerve injury models, non-human primate spinal nerve root compression models) (Ding et al., 2017). Such models better replicate human neuroanatomy and pain responses while enhancing preclinical pharmacodynamic predictability, providing robust platforms for developing targeted therapies.
4. Future exosome research should prioritize engineering exosomes specifically for drug delivery and clinical efficacy validation. Large-scale, multi-center studies with sufficient sample diversity and extended follow-up durations are essential to substantiate therapeutic efficacy and biosafety profiles (Figure 2).

## 4 Conclusion

Neuropathic pain (NP) is a refractory disorder involving multiple pathological mechanisms. It presents new therapeutic opportunities through the regulatory efficacy of miRNAs. Exosomes serve as ideal miRNA carriers due to their endogenous stability and targeted delivery advantages. Preclinical evidence confirms exosome-mediated miRNA delivery effectively alleviates NP by modulating core signaling pathways. However, clinical translation faces persistent challenges including exosomal heterogeneity, delivery efficiency bottlenecks, and complexity of personalized treatments. Addressing these requires multidisciplinary convergence of exosome engineering, biomaterials science, and clinical validation to accelerate reliable therapeutic solutions. Although current clinical implementation remains nascent, ongoing research strongly supports their translational potential.

## Glossary

Key terminology
miRNA: microRNA
NP: Neuropathic pain
Cells & exosomes
ADSC-EXs: Adipose-derived mesenchymal stem cell exosomes
BMSC-EVs: Bone marrow mesenchymal stem cell-derived extracellular vesicles
DRG: Dorsal root ganglion
EVs: Extracellular vesicles
huc-MSCs: Human umbilical cord mesenchymal stem cells
MSC: Mesenchymal stem cell
SKP-SCs: Skin-derived precursor Schwann cells
Clinical terms
AEs: Adverse events
ASIA: American Spinal Injury Association
BBB: Blood-brain barrier
CNS: Central nervous system
GMP: Good manufacturing practice
MCID: Minimal clinically important difference
Disease models
CCI: Chronic constriction injury
CIP: Chemotherapy-induced peripheral neuropathy
DPN: Diabetic peripheral neuropathy
ONI: Optic nerve injury
OXA: Oxaliplatin
pSNL: Partial sciatic nerve ligation
SCI: Spinal cord injury
SNI: Spared nerve injury
SNL: Spinal nerve ligation
TN: Trigeminal neuralgia
Molecular pathways
Akt: Protein kinase B
BDNF: Brain-derived neurotrophic factor
EFNA3: Ephrin-A3
GDNF: Glial cell-derived neurotrophic factor
GAP-43: Growth-associated protein 43
IRAK1: Interleukin-1 receptor-associated kinase 1
JNK3: c-Jun N-terminal kinase 3
KLF4: Krüppel-like factor 4
LAMP2B: Lysosome-associated membrane protein 2B
MAPK: Mitogen-activated protein kinase
mTOR: Mechanistic target of rapamycin
NF-κB: Nuclear factor kappa-light-chain-enhancer of activated B cells
NGF: Nerve growth factor
NLRP3: NLR Family pyrin domain containing 3
NMDA: N-Methyl-D-aspartate receptor
NT-3: Neurotrophin-3
PI3K: Phosphoinositide 3-kinase
PTEN: Phosphatase and tensin homolog
Rab27a/b: Ras-related protein Rab-27A/B
SEPT9: Septin 9
STAT3: Signal transducer and activator of transcription 3
TGF-β1: Transforming growth factor beta 1
TLR4: Toll-like receptor 4
TRAF6: TNF receptor-associated factor 6
TRPA1: Transient receptor potential ankyrin 1
TRPV1: Transient receptor potential vanilloid 1
TXNIP: Thioredoxin-interacting protein
ZEB1: Zinc Finger E-box binding homeobox 1
Other key terminology
DAMPs: Damage-associated molecular patterns
lncRNA: Long non-coding rna
TDN: Tetrahedral DNA nanostructure
UCA1: Urothelial cancer-associated 1
Technical methods
ESCRT: Endosomal sorting complexes required for transport
GWAS: Genome-wide association study
ISEV: International Society for Extracellular Vesicles
MISEV: Minimal information for EV studies
NGS: Next-generation sequencing
SCS: Spinal cord stimulation
TENS: Transcutaneous electrical nerve stimulation
