# Correction: Exosome-mediated miRNA delivery: a molecular switch for reshaping neuropathic pain therapy

**DOI:** 10.3389/fnmol.2025.1668038

**Published:** 2025-08-21

**Authors:** Ziqing Wei, Chunhui Guo, Hang Zhou, Yanling Wu, Xudong Zhou, Jibing Chen, Fujun Li

**Affiliations:** ^1^Graduate School, Guangxi University of Chinese Medicine, Nanning, China; ^2^Ruikang Hospital Affiliated to Guangxi University of Chinese Medicine, Nanning, China; ^3^Graduate School, Guangxi University, Nanning, China; ^4^Zhuhai Maternal and Child Health Care Hospital, (Guangxi University of Chinese Medicine), Zhuhai, China

**Keywords:** neuropathic pain, targeted delivery, miRNA therapeutics, exosomes, pain management, clinical translation

In the published article, the affiliation number 2 was erroneously assigned to Fujun Li in the author list. This affiliation number has now been removed from Fujun Li's assigned affiliations. The corrected affiliation appears below:

Fujun Li^4*^

^4^Zhuhai Maternal and Child Health Care Hospital, (Guangxi University of Chinese Medicine), Zhuhai, China

The original version of this article has been updated.

